# Synthesis and Characterization of High-Energy *Anti*-Perovskite Compounds Cs_3_*X*[B_12_H_12_] Based on Cesium Dodecahydro-*Closo*-Borate with Molecular Oxoanions (*X*^−^ = [NO_3_]^−^, [ClO_3_]^−^ and [ClO_4_]^−^)

**DOI:** 10.3390/molecules29020382

**Published:** 2024-01-12

**Authors:** Rouzbeh Aghaei Hakkak, Ioannis Tiritiris, Thomas Schleid

**Affiliations:** Institute for Inorganic Chemistry, University of Stuttgart, D-70569 Stuttgart, Germany; rouzbeh.aghaei-hakkak@iac.uni-stuttgart.de (R.A.H.); tiritiris@iac.uni-stuttgart.de (I.T.)

**Keywords:** *anti*-perovskite structure, hydroborates, high-energy materials, X-ray crystallography

## Abstract

Three novel *anti*-perovskite compounds, formulated as Cs_3_*X*[B_12_H_12_] (*X^−^* = [NO_3_]^−^, [ClO_3_]^−^, and [ClO_4_]^−^), were successfully synthesized through the direct mixing of aqueous solutions containing Cs_2_[B_12_H_12_] and Cs*X* (*X^−^*: [NO_3_]^−^, [ClO_3_]^−^, [ClO_4_]^−^), followed by isothermal evaporation. All three compounds crystallize in the orthorhombic space group *Pnma*, exhibiting relatively similar unit-cell parameters (e.g., Cs_3_[ClO_3_][B_12_H_12_]: *a* = 841.25(5) pm, *b* = 1070.31(6) pm, *c* = 1776.84(9) pm). The crystal structures were determined using single-crystal X-ray diffraction, revealing a distorted hexagonal *anti*-perovskite order for each. Thermal analysis indicated that the placing oxidizing anions *X*^−^ into the 3 Cs^+^ + [B_12_H_12_]^2−^ blend leads to a reduction in the thermal stability of the resulting *anti*-perovskites Cs_3_*X*[B_12_H_12_] as compared to pure Cs_2_[B_12_H_12_], so thermal decomposition commences at lower temperatures, ranging from 320 to 440 °C. Remarkably, the examination of the energy release through DSC studies revealed that these compounds are capable of setting free a substantial amount of energy, up to 2000 J/g, upon their structural collapse under an inert-gas atmosphere (N_2_). These three compounds represent pioneering members of the first ever *anti*-perovskite high-energy compounds based on hydro-*closo*-borates.

## 1. Introduction

The perovskite crystal structure, with its distinctive *ABX*_3_ composition, has long been a focal point in materials science [1] owing to its diverse range of applications, spanning from photovoltaics [2] to superconductors [3]. In recent years, a counterintuitive class of materials, referred to as *anti*-perovskites, has emerged as a promising avenue for scientific exploration [4]. Unlike their traditional perovskite counterparts, *anti*-perovskite materials exhibit a unique crystallographic arrangement, characterized by an inverted cation–anion order. This unconventional configuration imparts distinct electronic, optical, and structural properties, sparking significant interest in harnessing their potential for groundbreaking advancements in various technological domains. The exploration of *anti*-perovskite materials has gained momentum due to their exceptional properties; these materials have demonstrated promise in diverse applications, such as having a high ionic conductivity [5], as well as opto-electronic [6], catalytic [7] and thermoelectric applications [8].

Boron (B) as a non-metal fuel has garnered considerable attention in advanced solid propellants. With a mass calorific value of up to 58.7 MJ/kg [9,10], boron surpasses another typical metal fuel, such as aluminum (Al), by almost two times, since aluminum has a calorific value of 30.8 MJ/kg. Moreover, the volumetric calorific value of boron reaches an impressive 135.2 kJ/cm^3^, significantly exceeding that of Al (83.3 kJ/cm^3^) by 51.9 kJ/cm^3^ [11,12]. Boron-based, fuel-rich propellants typically comprise the non-metal fuel boron, oxidants, binders, and additives. Serving as the pivotal component in propellants with a high mass ratio (usually ranging from 25 to 40%), the non-metal fuel boron continues to be a prominent focus in ongoing research [13,14].

On the other hand, hydroborates, notably with the dodecahydro-*closo*-dodecaborate anion [B_12_H_12_]^2−^, have been recognized since 1960 [15]. The alkyl-ammonium and alkali-metal compounds and derivatives originating from these hydroborates have captured the attention of numerous researchers owing to their distinctive properties [16]. Alkali- and alkaline-earth metal compounds containing the [B_12_H_12_]^2−^ anion have exhibited outstanding stability over a wide temperature range. As an example, Cs_2_[B_12_H_12_] with an *anti*-fluorite structure arrangement remains in a solid and stable state up to temperatures of approximately 800 °C [17].

*Anti*-perovskites incorporating the dodecahydro-*closo*-dodecaborate anion [B_12_H_12_]^2−^ have been previously synthesized, with a number of examples including Cs_3_Cl[B_12_H_12_], (NH_4_)_3_Br[B_12_H_12_] [18], K_3_I[B_12_H_12_] [19], Cs_3_[BH_4_][B_12_H_12_] [20], and luminescent Tl_3_Cl[B_12_H_12_] [21]. Among these compounds, only Tl_3_Cl[B_12_H_12_] exhibits a hexagonal and not a cubic *anti*-perovskite structure.

Energetic materials are one of the main topics in chemistry, because of their wide range of application as explosives [22], propellants [23] and pyrotechnic materials [24]. Boron-cluster compounds, specifically [B_12_H_12_]^2−^-containing ones, have become a remarkable subject of energetic research. The increased stability of these compounds compared to other borates, coupled with their substantial hydrogen and boron content, positions them at the forefront of energetic investigations. The amalgamation of the [B_12_H_12_]^2−^ anion with energetic yet less stable cations has led to the creation of energetic salts, exemplified by diguanidinium [25] and dihydrazinium salts [26]. Under inert atmospheres like N_2_, dihydrazinium salts have demonstrated the capacity to release approximately 1650 J/g of heat next to elemental hydrogen (H_2_). Meanwhile, the combustion of diguanidinium salts has been shown to unleash an extraordinary amount of energy, reaching up to 34 kJ/g [27].

Perovskite chemistry recently advanced itself toward the synthesis of new types of energetic materials. A variety of different types of these energetic perovskites were syntheses with different compositions, such as compounds based on inorganic [28] and organic materials [29,30]. These compounds mostly use oxidizing anions, such as perchlorate, on their *X*-site in an *ABX*_3_ perovskite, but there is no record of energetic *anti*-perovskites to date.

In this research, we focused on the synthesis of the first ever high-energy *anti*-perovskite compounds and add new members to this family of high energetic materials. Three novel *anti*-perovskite compounds, namely Cs_3_[NO_3_][B_12_H_12_] (**I**), Cs_3_[ClO_3_][B_12_H_12_] (**II**) and Cs_3_[ClO_4_][B_12_H_12_] (**III**), were successfully synthesized and comprehensively characterized using various methods, include X-ray, single-crystal structure analysis, vibrational spectroscopy (Raman), thermogravimetry (TG), and differential scanning calorimetry (DSC).

## 2. Results and Discussion

### 2.1. Crystallography

**Figure 1:**
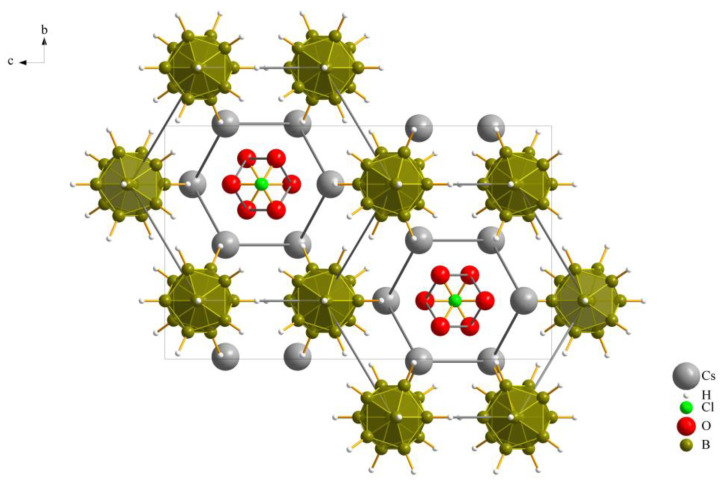
Extended unit cell content of the crystal structure of Cs_3_[ClO_3_][B_12_H_12_] (**II**) as viewed along [100].

The three new compounds Cs_3_*X*[B_12_H_12_] crystallize similarly in the orthorhombic space group *Pnma* with four formula units per unit cell and have similar cell parameters. Details of the crystallographic data and their determination are provided in Table 1 and an overall look at the unit cell of Cs_3_[ClO_3_][B_12_H_12_] (**II**), as an example, is depicted in Figure 1.

In these three compounds under consideration, four distinct molecular polyatomic anions are present: nitrate [NO_3_]^−^, chlorate [ClO_3_]^−^, perchlorate [ClO_4_]^−^ and dodecahydro-*closo*-dodecaborate [B_12_H_12_]^2−^. Each of these species exhibits standard bond lengths and angles. Specifically, for nitrate, chlorate and perchlorate, the N–O and Cl–O distances and angles fall within the approximate ranges of 125–127 pm and 118–119°, 146–147 pm and 107–108°, and 141–145 pm and 109–110°, respectively [31,32,33]. Additionally, the B–B bond lengths within the [B_12_H_12_]^2−^ anions typically range between 174 and 181 pm, and are consistently observed [25,26] for all three cases, and the same holds for the B–H distances (108–117 pm).

In all three compounds, the *quasi*-icosahedral [B_12_H_12_]^2−^-cluster units are bisected by both a mirror plane and a glide plane perpendicular to each other. All Cs^+^ cations exhibit a consistent placement on glide planes, while the (Cs1)^+^ cations, in addition to the glide planes, also reside on a mirror plane. Likewise, nitrogen and chlorine within these compounds undergo this bisection using a perpendicular mirror and glide planes.

**Figure 2:**
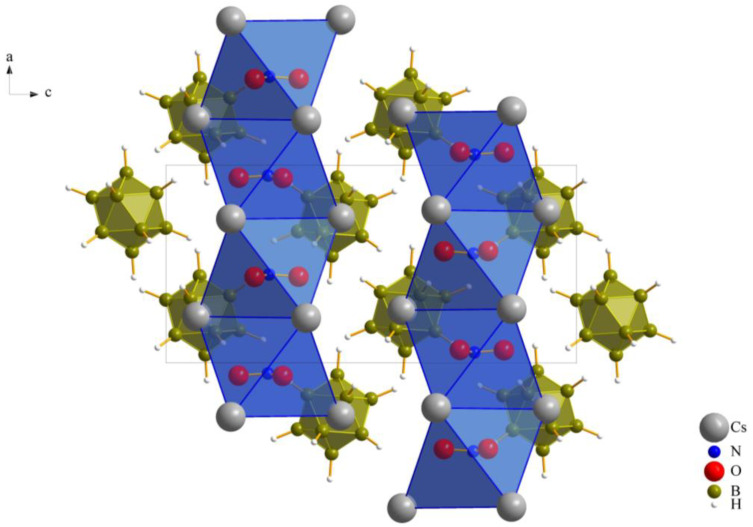
Hexagonal *anti*-perovskite ordering of Cs_3_[NO_3_][B_12_H_12_] (**I**) as viewed along [010].

As previously noted, these compounds exhibit a unique combination reminiscent of the *anti*-perovskite *A*_3_*XB* structure (*A*^+^ = Cs^+^, *X*^−^ = [NO_3_]^−^, [ClO_3_]^−^ and [ClO_4_]^−^, *B*^2−^ = [B_12_H_12_]^2−^). However, it is noteworthy that, despite this association, the crystalline arrangement observed conforms to the hexagonal *anti*-perovskite pattern in all cases. In this specific configuration, the complex molecular oxoanions occupying the *X*^−^ site are enveloped by six cations from the *A^+^* site, forming infinite pillars in the present compounds by sharing *trans*-oriented faces. These pillars are parallel to the crystallographic [100] axis, as an example this arrangement in compound (**I**), as depicted in Figure 2, and are bundled like the hexagonal close packing of rods, as emphasized in Figure 1 for compound (**II**).

**Figure 3:**
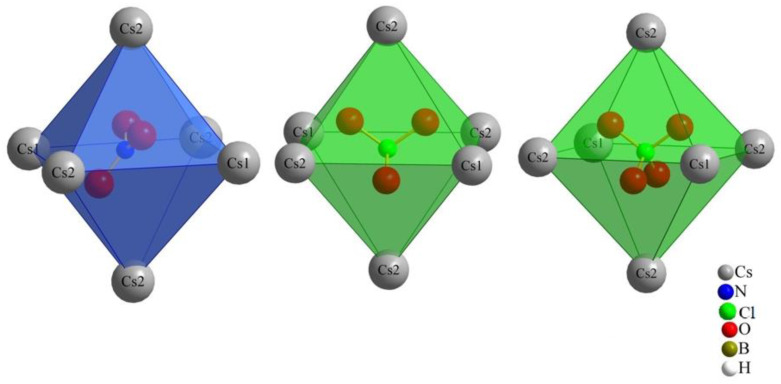
Orientation of the monovalent oxoanions in compounds (**I**), (**II**) and (**III**).

The monovalent molecular anions, nitrate, chlorate and perchlorate, exhibit a distinctive three-dimensional positioning within the octahedral coordination environment with Cs^+^ cations (two (Cs1)^+^ and four (Cs2)^+^), leading to their considerable structural resemblance. Our initial prediction for nitrate [NO_3_]^−^, based on its triangular planar two-dimensional shape, differed from that for chlorate and perchlorate, both featuring three-dimensional shapes (trigonal pyramidal and tetrahedral, respectively). However, the key to their similarity lies within the octahedral Cs^+^-cation arrangement. As illustrated in Figure 3, the nitrate anion is not centrally located within this octahedron, neither parallel nor perpendicular to the square. Instead, it assumes an oblique position. The standard deviation percentages (SDP) for compounds (**I**), (**II**) and (**III**) are 4.52, 1.13 and 1.12, respectively. These values suggest that chlorate and perchlorate can realize nearly ideal (Cs^+^)_6_ octahedra around them. In contrast, nitrate introduces a subtle distortion to the octahedral geometry. This nuanced distortion emphasizes the intricacies associated with the positioning of the nitrate anion within the crystal structure, contributing to the slight structural variations observed among the three compounds.

The coordination sphere around the Cs^+^ cations for both Cs1 and Cs2 in all three compounds consists of twelve (or even more) atoms, which is similar to the “normal” coordination numbers for the *A* site in cubic or hexagonal *ABX*_3_ perovskites. The cesium cations (1 and 2) are surrounded by two monovalent oxoanions (nitrate, chlorate and perchlorate) and four divalent anions ([B_12_H_12_]^2−^) with a *cis*-orientation, so the cesium cations are connected with two bonds and one bond to the oxygen atoms of the monovalent anions, and with one triple contact and three double contacts to surrounding boron cluster anions [B_12_H_12_]^2−^.

**Figure 4:**
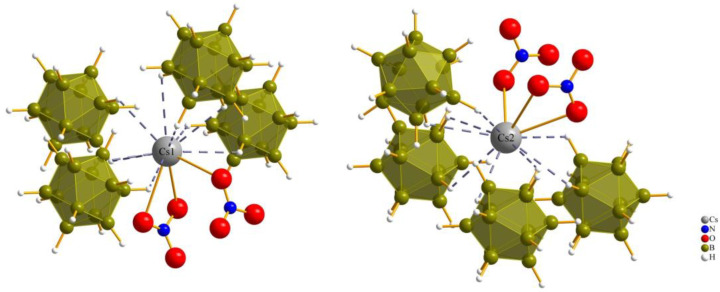
Anionic coordination spheres around (Cs1)^+^ and (Cs2)^+^ in Cs_3_[NO_3_][B_12_H_12_] (**I**).

**Figure 5:**
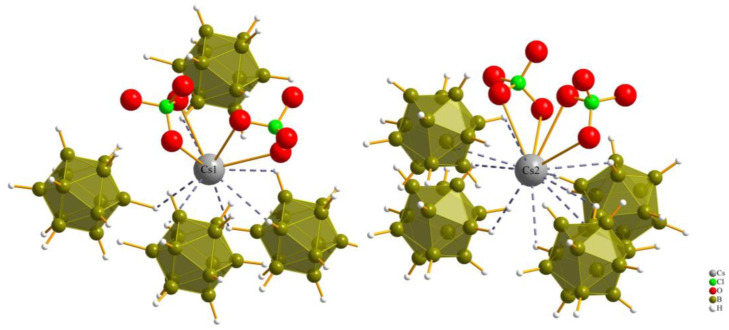
Anionic coordination spheres around (Cs1)^+^ and (Cs2)^+^ in Cs_3_[ClO_4_][B_12_H_12_] (**III**).

The coordination environment around both Cs^+^ cations (Cs1 and Cs2) in all three compounds is consistent with a coordination number of 12 and aligns well with the typical coordination numbers for perovskites (Figure 4 and Figure 5).

For compound (**III**), each of the cesium cations is connected again to two perchlorate anions and four boron-cluster anions, but in this compound each anion has two instances of contact with each cesium cation to add up to the coordination number of 12 (Figure 5).

Both kinds of cesium cations establish two bidentate Cs–O contacts to two monovalent perchlorate oxoanions, while (Cs1)^+^ is connected to four boron-cluster anions with different numbers of Cs–H contacts to each, with one monodentate, two bidentate and one tridentate contact and, for (Cs2)^+^, the number of Cs–H contacts to the four neighboring boron-cluster anions is ordered differently. Two monodentate and two bidentate contacts are used to complete the *anti*-perovskite twelvefold coordination for this position too. The interatomic distances for Cs–H and Cs–O bonds are listed in Table 2.

**Figure 6:**
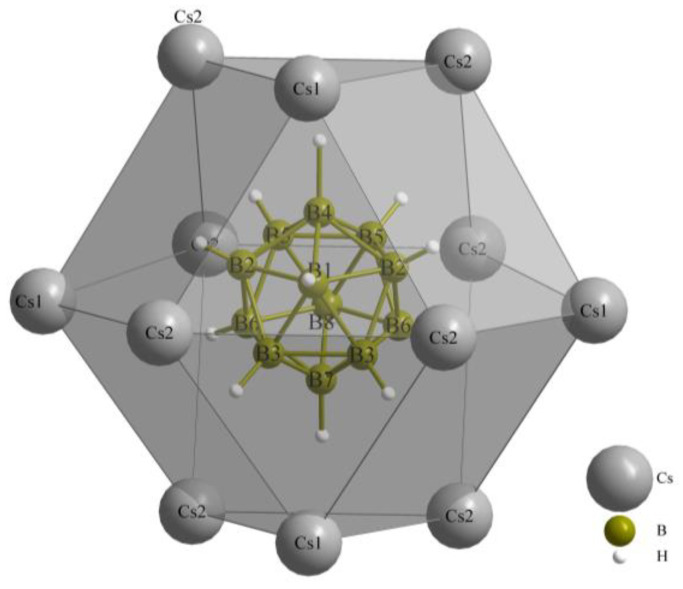
*Anti*-cuboctahedron {[B_12_H_12_](Cs1)_4_(Cs2)_8_}^10+^ as result of the mixed hexagonal close packing of Cs^+^ cations and [B_12_H_12_]^2−^ anions.

Figure 6 illustrates the *anti*-cuboctahedron {[B_12_H_12_](Cs1)_4_(Cs2)_8_}^10+^, which is formed by the mixed hexagonal close packing of Cs^+^ cations and [B_12_H_12_]^2−^ anions. This arrangement follows the ∞3{([B_12_H_12_]Cs_12/4_)^+^} structure, which is suitable for hosting X^−^-anion chains along [100], embedded into ∞1{[*X*Cs_6/2_]^2+^} pillars.

Despite its structure description and the cubic close packing of [B_12_H_12_]^2−^ anions with Cs^+^ cations in all tetrahedral voids, the *anti-*fluorite arrangement of Cs_2_[B_12_H_12_] (*V*_m_ = 216.15 cm^3^/mol) appears rather fluffy. Therefore, the formation of double salts with the composition Cs_3_*X*[B_12_H_12_] is regarded as having derived from the *pseudo-*binaries Cs*X* and Cs_2_[B_12_H_12_], and always occurs under densification when compared to the sum of their molar volumes (Σ = *V*_m_(Cs_2_[B_12_H_12_]) + *V*_m_(Cs*X*); see Table 3). Even the formation of the dihydrate Cs_2_[B_12_H_12_] · 2 H_2_O matches this scheme.

The Goldschmidt tolerance factor is a widely employed tool to assess the stability of perovskites, calculated using the formula t_G_ = (*rB* + *rA*)/√2(*rX* + *rA*), where *rA*, *rB*, and *rX* represent the ionic radii of *A*, *B*, and *X*, respectively, and an ideal perovskite *ABX*_3_ has a tolerance factor of 1 [21]. Calculating the Goldschmidt tolerance factors (t_G_) with *r*([B_12_H_12_]^2−^) = 322 pm, *r*(Cs^+^) = 177 [21], and *r*([NO_3_]^−^), *r*([ClO_3_]^−^) and *r*([ClO_4_]^−^) as 200, 208 and 225 [42], respectively, yields values of 0.936, 0.917 and 0.878 for compounds (**I**)–(**III**), respectively. These values fall within a narrow range, indicating a trend that aligns with predictions for the same perovskite-type structure, but not the hexagonal one. This seems to be often the case for *anti*-perovskite-type cesium compounds and was previously stated for Cs_3_OCl [43], Cs_3_OBr, Cs_3_OI [44] and Cs_3_OAu [45], where O^2−^ plays the role of *X*^−^ and Cl^−^ takes the [B_12_H_12_]^2−^ part.

### 2.2. Raman Spectroscopy

Raman spectroscopy was executed on powder samples of the compounds (**I**), (**II**) and (**III**). All three compounds exhibit similarities in four distinct regions associated with the vibrational characteristics of the dianionic boron cluster [B_12_H_12_]^2−^. In the spectral range spanning from 2500 to 2400 cm^−1^, a distinct manifestation of the symmetric breathing vibration of the boron cage becomes apparent. This phenomenon is predominantly influenced by the stretching mode of B–H bonds. Notably, the cubic Cs_2_[B_12_H_12_] crystals in the space group *Fm*$\overset{-}{3}$ [46] show only one peak in the specified region. Intriguingly, when the compound symmetry is diminished to orthorhombic in the space group *Pnma*, an unique outcome emerges—the region undergoes splitting, introducing a distinctive spectral pattern [21]. Accordingly, the anticipated finding is the observation of just two discernible peaks. Another marked region, spanning from 950 to 920 cm^−1^, corresponds to B–B stretching vibrations. At 750 cm^−1^, the most intense peak signifies the symmetric breathing mode of the B_12_ cage, while the interval from 570 to 560 cm^−1^ can be ascribed to more B–B stretching vibrations, culminating in lattice vibrations at approximately 200 cm^−1^.

**Figure 7:**
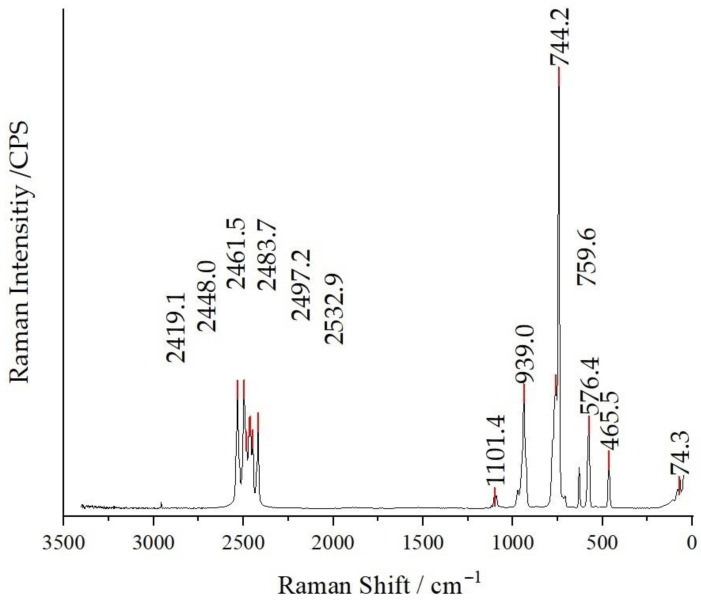
Raman spectrum of Cs_3_[ClO_3_][B_12_H_12_] (**II**) recorded at an excitation wavelength of λ = 740 nm.

In the case of compound (**I**), a subtle peak at 1350 cm^−1^ is discernible, which is attributable to the asymmetric stretch of the nitrate group. Notably, an intense symmetric stretching mode of the nitrate anion can be observed around 1050 cm^−1^, accompanied by a less pronounced peak at 700 cm^−1^, corresponding to its in-plane deformation mode. For compounds (**II**) and (**III**), distinctive features emerge, including three regions at 950 cm^−1^ associated with symmetric Cl–O stretching (overlapping with B–B skeleton vibrations), and wavenumbers of 620 and 470 cm^−1^, indicative of the active and inactive deformations of the Cl–O bonds, respectively [47]. As an example, the Raman spectrum of compound (**II**) is depicted in Figure 7, and the Raman spectra of compounds (**I**) and (**III**) are available as Figures S1 and S2 in the Supplementary Material.

### 2.3. Thermal Analysis

**Figure 8:**
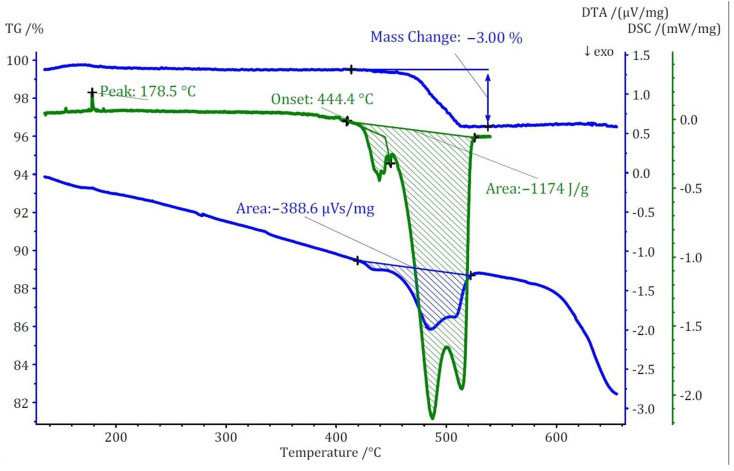
Thermoanalytical TG/DTA and DSC diagram of Cs_3_[NO_3_][B_12_H_12_] (**I**).

Two distinct methods of thermal analyses, namely TG/DTA and DSC, were conducted for the three compounds under investigation. The TG analysis revealed that the thermal decomposition of these compounds occurs at significantly lower temperatures when compared to the pure cesium salt of [B_12_H_12_]^2−^, namely cubic Cs_2_[B_12_H_12_]. In terms of mass loss, compound (**I**) exhibits a single-step thermal decomposition with an onset point at approximately 440 °C, resulting in a 3% mass loss (Figure 8). Conversely, compounds (**II**) and (**III**) undergo thermal decomposition in two closely spaced steps, each with less than 1% mass loss for both. The onset points for these steps are 360 °C for compound (**II**) and 320 °C for compound (**III**). The mass loss for these compounds initiates at substantially lower temperatures compared to Cs_2_[B_12_H_12_], which decomposes at around 800 °C.

**Figure 9:**
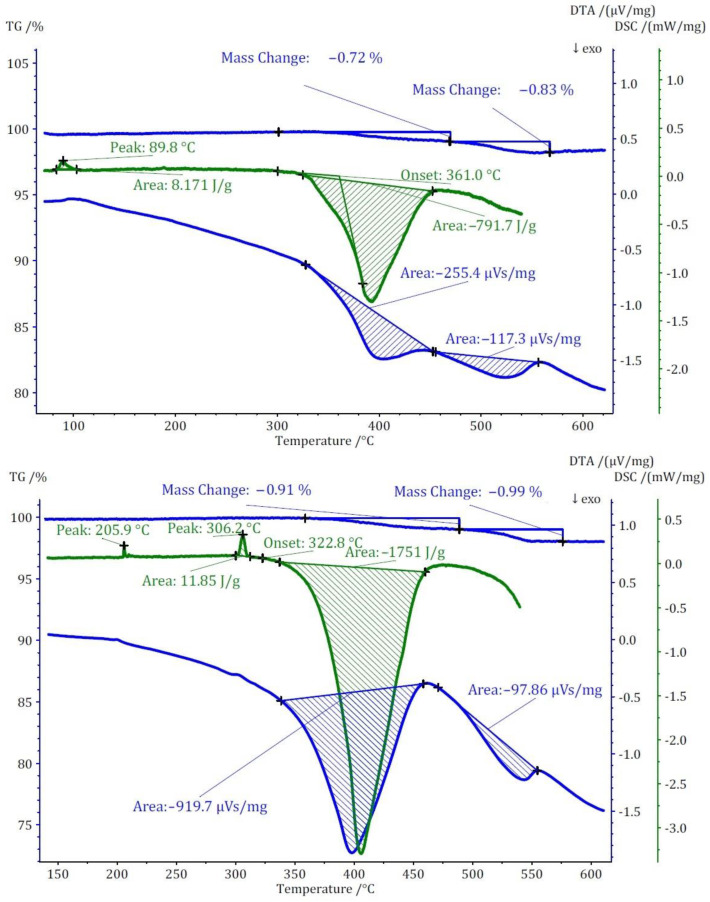
Thermoanalytical TG/DTA and DSC diagrams of Cs_3_[ClO_3_][B_12_H_12_] (**II**) (**top**) and Cs_3_[ClO_4_][B_12_H_12_] (**III**) (**bottom**).

The thermal study of these compounds also revealed intriguing energetic properties, particularly through their DSC analyses. Compound (**I**) exhibits a small endothermic peak around 180 °C, likely associated with a phase transition due to there being no observable mass change at this temperature. Subsequently, a substantial exothermic peak emerges at around 440 °C with an area of −1175 J/g. For compound (**II**), an endothermic peak appears at a remarkably low temperature of around 90 °C, followed by two consecutive exothermic peaks. The first exothermic peak has an area of approximately 800 J/g. Unfortunately, the DSC analysis could not be extended beyond 550 °C due to the crucible limitations. However, by comparing DTA and DSC numbers for the first peak, the area of the second peak was estimated to be approximately −360 J/g. Collectively, compound (**II**) exhibits an energy release of around 1150 J/g in two steps. In the case of compound (**III**), two endothermic peaks without a change in mass are observed at around 206 and 306 °C, followed by one substantial and one small exothermic peak. The first exothermic peak is observable in both DSC and DTA analyses with an energy release of around 1750 J/g. The second peak, detectable only in the DTA analysis, has an estimated energy release of around 200 J/g, resulting in a total energy release of approximately 1950 J/g (Figure 9). The observed phase transitions at lower temperatures align with findings from prior thermal studies on similar [B_12_H_12_]^2−^-containing compounds [10,11]. Earlier investigations into Cs_2_[B_12_H_12_] [17] demonstrated the remarkable stability of the cesium salt, maintaining its structural integrity up to approximately 800 °C, and, during the thermal decomposition, a singular endothermic peak was identified. However, the addition of oxidizing anions into the compound induced a notable shift, causing decomposition to initiate at far lower temperatures. This deviation from the behavior of simple salts was marked by the emergence of strongly exothermic decomposition reactions.

The decomposition temperatures of these three compounds exceed those of traditional energetic materials by far, rendering them exceptionally thermally stable. Notably, the heat release of compound (**III**) is nearly equivalent to that one of 1,3,5,7-tetranitro-1,3,5,7-tetrazocane (HMX, 1987 J/g). Refer to Table 4 for a comprehensive depiction of the decomposition temperatures of compounds (**I**)–(**III**) alongside the traditional high-energy materials and their density [48]. Density is a pivotal parameter in the high-energy materials domain and a higher density always favors these materials due to their lower space consumption and increased ratio of energy per volumetric space. The density of compounds (**I**) to (**III**) are relatively equal, ranging from 2.56 to 2.69 g·cm^−3^, respectively, due to their similarity in both unit-cell parameter and molar masses, and they always show a denser packing in comparison to pure Cs_2_[B_12_H_12_], as mentioned in a previous section.

## 3. Materials and Methods

### 3.1. Synthesis

The three *anti*-perovskite compounds featuring dodecahydro-*closo*-dodecaborate anions were synthesized by dissolving Cs_2_[B_12_H_12_] (ABCR (Karlsruhe, Germany)) in a minimal amount of distilled water. Subsequently, a 1:1 ratio of appropriate cesium salts (cesium nitrate: Sigma Aldrich (St. Louis, MO, USA), cesium chlorate: mixture of sodium chlorate (Fluka (Buchs, Switzerland)) and cesium hydroxide (Sigma Aldrich) and cesium perchlorate: prepared by mixing freshly obtained cesium hydroxide (Sigma Aldrich) with perchloric acid (70%, Fisher Scientific (Hampton, NH, USA))) was added to portions of this solution. The resulting clear brine was subjected to isothermal evaporation at room temperature, yielding transparent and colorless single crystals of the respective salts Cs_3_*X*[B_12_H_12_] (*X^−^* = [NO_3_]^−^, [ClO_3_]^−^ and [ClO_4_]^−^).

### 3.2. Devices

#### 3.2.1. X-ray Crystallography

Single-crystal X-ray diffraction data were acquired utilizing a Nonius κ-CCD diffractometer with Mo-Kα radiation graphite-monochromatized to a wavelength of λ = 71.07 pm. The entire process, spanning initial structure solutions via direct methods to subsequent structure refinements, was conducted using the SHELX-97 program package [49,50]. Atomic positions were accurately determined through difference Fourier maps based on the collected data, facilitating the anisotropic refinement of all non-hydrogen atoms. Detailed information regarding the data collection and the outcomes of the final structure refinements is provided in Table 1. Supplementary data related to the structure refinements can be accessed through the Fachinformationszentrum (FIZ) Karlsruhe (D-73644 Eggenstein-Leopoldshafen, E-mail: crysdata@fiz-karlsruhe.de) and are referenced with the numbers outlined in Table 1. Fractional atomic positions are additionally available in Tables S1–S3 of the Supplementary Material. It is noteworthy that, in this study, hydrogen atom positions were ascertained through electron-density maps generated by difference Fourier syntheses utilizing the X-ray diffraction data. No further calculations or corrections were deemed necessary, given that the positions of these hydrogen atoms are well-defined based on their precise angles and distances in relation to other atoms.

#### 3.2.2. Thermal Analysis

Two distinct instruments were employed for thermal analyses in this study. The first device, STA 449 C Jupiter by Netzsch (Selb, Germany) with alumina crucibles, played a crucial role in the investigation. TG analysis was carried out in alumina crucibles with a heat rate of 5 K min^−1^ under an argon atmosphere. The second device, DSC 201, also from Netzsch, operated as a differential scanning calorimeter with a heating rate of 5 K min^−1^ and a nitrogen flow of 20 mL min^−1^ and samples prepared in aluminum crucibles with a maximum temperature of 600 °C. This instrument was essential in the research, providing precise measurements of the various thermal properties of the samples. These properties encompassed heat capacity, energy changes and activation energies, all of which were indispensable for the comprehensive analysis conducted in the study.

#### 3.2.3. Raman Spectroscopy

We performed Raman spectroscopic analyses with the DXR Smart Raman system, a product developed by Thermo Fisher (Waltham, MA, USA), which operates at an excitation wavelength of λ = 740 nm. The spectroscopic data collected during these analyses were meticulously scrutinized.

## 4. Conclusions

The synthesis of three novel *anti*-perovskite compounds, Cs_3_*X*[B_12_H_12_] (*X^−^* = [NO_3_]^−^, [ClO_3_]^−^, [ClO_4_]^−^), is presented in this research. These compounds exhibit orthorhombic crystal structures in the space group *Pnma*, with comparable unit-cell parameters. Single-crystal X-ray diffraction has unveiled a hexagonal *anti*-perovskite order for each compound, characterized by pillars ${\;}_{\infty }^{1}${[*X*Cs_6/2_]^2+^} of *trans*-faced connected [*X*Cs_6_]^5+^ octahedra, which are bundled like a hexagonal close rod packing, held together by *quasi*-icosahedral [B_12_H_12_]^2−^ anions with a counter-intuitive Goldschmidt tolerance factor far below 1. Thermal analyses have revealed a reduction in thermal stability as compared to Cs_2_[B_12_H_12_], with decomposition processes commencing at lower temperatures (from 320 to 440 °C) upon the introduction of oxidizing anions. Additionally, differential scanning calorimetry (DSC) has indicated that the presence of molecular oxoanions in the compounds leads to an exceptional energy release potential, reaching up to 2000 J/g, as the compounds undergo structural collapse in an inert-gas atmosphere (N_2_). The substantial energy release and elevated decomposition characteristics render these compounds promising candidates for thermally stable energetic materials. This pioneering advancement solidifies their significance in the domain of energetic *anti*-perovskite materials.

## Figures and Tables

**Table 1:** Crystallographic data for the crystal structures of Cs_3_[NO_3_][B_12_H_12_] (**I**), Cs_3_[ClO_3_][B_12_H_12_] (**II**) and Cs_3_[ClO_4_][B_12_H_12_] (**III**).

Compound	Cs_3_[NO_3_][B_12_H_12_] (I)	Cs_3_[ClO_3_][B_12_H_12_] (II)	Cs_3_[ClO_4_][B_12_H_12_] (III)
temperature, K	293	293	293
crystal system	orthorhombic	orthorhombic	orthorhombic
space group	*Pnma*	*Pnma*	*Pnma*
*a*, pm	848.51(5)	841.25(5)	892.46(5)
*b*, pm	1045.62(6)	1070.31(6)	1054.89(6)
*c*, pm	1761.38(9)	1776.84(9)	1718.53(9)
*D*_cal_, g cm^−3^	2.561	2.591	2.627
*μ*(MoK_α_), mm^−1^	6.95	6.95	6.88
*F*(000), e^−^	1072	1112	1144
*hkl* range	12, 15, 25	10, 13, 22	11, 13, 22
2*θ*_max_, deg	31.51	27.48	27.48
refl. measured	31,742	27,436	29,173
refl. unique	2730	1929	1956
*R*_int_/*R_σ_*	0.064, 0.026	0.117, 0.042	0.055, 0.021
*R*_1_/*wR*_2_	0.042, 0.116	0.050, 0.095	0.022, 0.048
GooF	1.085	1.061	1.088
CSD number	2314128	2313882	2313868

**Table 2:** Cs–O and Cs–H bond lengths in compounds (**I**), (**II**) and (**III**).

Compound	Cs–O Bond Lengths (*d*/pm)	Cs–H Bond Lengths (*d*/pm)
Cs_3_[NO_3_][B_12_H_12_] (**I**)	310–320	313–359
Cs_3_[ClO_3_][B_12_H_12_] (**II**)	318–327	307–355
Cs_3_[ClO_4_][B_12_H_12_] (**III**)	316–330	311–357

**Table 3:** Molar volumes (*V*_m_ in cm^3^·mol^−1^) of compounds (**I**) to (**III**), their precursor materials, and similar hydro-borate perovskites, with a comparison of the calculated sum of the components and the real values. Vm(B) represents V_m_(Cs_2_[B_12_H_12_]), and V_m_(C) represents V_m_(CsX) or V_m_ (ice-I), respectively.

Compound A	*V*_m_(A)	*V*_m_(B) + *V*_m_(C)	*V*_m_(B or C)	Component B and C
–	–	–	216.2	Cs_2_[B_12_H_12_] [19] (**B**)
Cs_3_[NO_3_][B_12_H_12_]	235.3	270.4	54.2	Cs[NO_3_] [34] (**C**)
Cs_3_[ClO_3_][B_12_H_12_]	240.9	273.1	56.9	Cs[ClO_3_] [35] (**C**)
Cs_3_[ClO_4_][B_12_H_12_]	243.6	275.5	69.3	Cs[ClO_4_] [36] (**C**)
Cs_3_Cl[B_12_H_12_] [18]	220.8	258.3	42.1	CsCl [37] (**C**)
Cs_3_Br[B_12_H_12_] [18]	224.8	263.2	47.0	CsBr [38] (**C**)
Cs_3_I[B_12_H_12_] [18]	236.9	273.4	57.2	CsI [39] (**C**)
Cs_2_[B_12_H_12_] · 2 H_2_O [40]	202.0	254.2	19.0	H_2_O (ice-I) [41] (**C**)

**Table 4:** Decomposition temperatures of compounds (**I**)–(**III**) as compared to some traditional energetic materials and a compilation of their densities.

Compound	Decomposition Peak (°C)	Density (g·cm^−3^)
Cs_2_[B_12_H_12_]	800	1.43
Cs_3_[NO_3_][B_12_H_12_] (**I**)	440–480–514	2.56
Cs_3_[ClO_3_][B_12_H_12_] (**II**)	393	2.59
Cs_3_[ClO_4_][B_12_H_12_] (**III**)	405	2.63
Hexahydro-1,3,5-trinitro-1,3,5-triazine (RDX)	210	1.82
1,3,5,7-Tetranitro-1,3,5,7-tetrazocane (HMX)	283	1.91
2,4,6-Trinitrotoluene (TNT)	290	1.65

## Data Availability

CIF files are available at the CSD database. Other raw files are not necessary, and all the diagram and picture represent in article file and the Supplementary Material.

## Supplementary Materials

The following supporting information can be downloaded at: https://www.mdpi.com/article/10.3390/molecules29020382/s1, Table S1. Atomic coordinates and coefficients of the equivalent isotropic displacement parameters for Cs_3_[NO_3_][B_12_H_12_] (**I**); Table S2. Atomic coordinates and coefficients of the equivalent isotropic displacement parameters for Cs_3_[ClO_3_][B_12_H_12_] (**II**); Table S3. Atomic coordinates and coefficients of the equivalent isotropic displacement parameters for Cs_3_[ClO_4_][B_12_H_12_] (**III**); Figure S1. Raman spectrum of Cs_3_[NO_3_][B_12_H_12_] (**I**); Figure S2. Raman spectrum of Cs_3_[ClO_4_][B_12_H_12_] (**III**).
