# Supplementary material for: Synthesis and Characterization of High-Energy Anti-Perovskite Compounds Cs3X[B12H12] Based on Cesium Dodecahydro-Closo-Borate with Molecular Oxoanions (X− = [NO3]−, [ClO3]− and [ClO4]−)
Source: Molecules. 2024 Jan 12;29(2):382. doi: 10.3390/molecules29020382 (PMC10820151; doi:10.3390/molecules29020382)
Supplement: Supplementary file 1 [file molecules-29-00382-s001.zip › molecules-2807993-supplementary.pdf]

Table S1. Atomic coordinates and coefficients of the equivalent isotropic displacement parameters for $Cs_3[NO_3][B_{12}H_{12}]$ (**I**).

Atom	*Wyckoff* site	*x / a*	*y / b*	*z / c*	U_{eq} / pm^2
Cs1	4*c*	0.26627(5)	$^1/_4$	0.57549(2)	434.2(14)
Cs2	8*d*	0.22713(4)	0.00310(2)	0.341697(17)	423.2(13)
N	4*c*	0.0534(7)	$^1/_4$	0.7513(3)	448(11)
O1	4*c*	0.0646(9)	$^1/_4$	0.8230(3)	786(17)
O2	8*d*	0.0661(8)	0.1473(5)	0.7156(3)	1008(17)
B1	4*c*	0.1646(7)	$^1/_4$	0.1788(3)	346(11)
H1	4*c*	0.116	$^1/_4$	0.237	416
B2	4*c*	0.0418(7)	$^1/_4$	0.0962(3)	329(11)
H2	4*c*	–0.088	$^1/_4$	0.100	395
B3	4*c*	0.4384(7)	$^1/_4$	0.0823(3)	304(11)
H3	4*c*	0.568	$^1/_4$	0.078	365
B4	4*c*	0.3180(7)	$^1/_4$	0.0006(3)	349(11)
H4	4*c*	0.368	$^1/_4$	–0.057	418
B5	8*d*	0.3395(5)	0.1649(4)	0.1556(2)	328(8)
H5	8*d*	0.404	0.110	0.199	393
B6	8*d*	0.3266(5)	0.1116(4)	0.0598(2)	345(8)
H6	8*d*	0.382	0.022	0.041	413
B7	8*d*	0.1549(5)	0.1116(4)	0.1195(2)	325 (8)
H7	8*d*	0.099	0.022	0.139	390
B8	8*d*	0.1411(5)	0.1648(4)	0.0231(2)	371(8)
H8	8*d*	0.077	0.110	–0.020	445

Table S2. Atomic coordinates and coefficients of the equivalent isotropic displacement parameters for $Cs_3[ClO_3][B_{12}H_{12}]$ (**II**).

Atom	*Wyckoff* site	*x / a*	*y / b*	*z / c*	U_{eq} / pm^2
Cs1	4*c*	0.26127(7)	$^1/_4$	0.57179 (4)	497(3)
Cs2	8*d*	0.23179(5)	0.00982(5)	0.34315(3)	475(2)
Cl	4*c*	0.0097(2)	$^1/_4$	0.75085(13)	470(6)
O1	4*c*	0.0740(9)	$^1/_4$	0.8277(4)	678(22)
O2	8*d*	0.0738(7)	0.1394(5)	0.7119(3)	717(17)
B1	4*c*	0.1668(11)	$^1/_4$	0.1770(6)	35(2)
H1	4*c*	0.119	$^1/_4$	0.235	422
B2	4*c*	0.0411(11)	$^1/_4$	0.0975(6)	394(22)
H2	4*c*	–0.089	$^1/_4$	0.103	469
B3	4*c*	0.4394(11)	$^1/_4$	0.0829(6)	360(23)
H3	4*c*	0.570	$^1/_4$	0.079	432
B4	4*c*	0.3179(11)	$^1/_4$	0.0011(6)	411(26)
H4	4*c*	0.369	$^1/_4$	0.944	493
B5	8*d*	0.3379(8)	0.1663(7)	0.1551(4)	350(17)
H5	8*d*	0.402	0.112	0.198	422
B6	8*d*	0.3259(8)	0.1155(7)	0.0600(4)	399(18)
H6	8*d*	0.382	0.028	0.041	478
B7	8*d*	0.1556(8)	0.1144(7)	0.1187(4)	419(19)
H7	8*d*	0.100	0.027	0.137	503
B8	8*d*	0.1412(8)	0.1666(7)	0.0229(4)	391(18)
H8	8*d*	0.076	0.113	–0.0201	469

Table S3. Atomic coordinates and coefficients of the equivalent isotropic displacement parameters for $Cs_3[ClO_4][B_{12}H_{12}]$ (**III**).

Atom	*Wyckoff* site	x / a	y / b	z / c	U_{eq} / pm^2
Cs1	4*c*	0.27314(3)	$^1/_4$	0.571528(17)	372.8(10)
Cs2	8*d*	0.22891(2)	0.007474(18)	0.342365(12)	342.1(9)
Cl	4*c*	0.01695(9)	$^1/_4$	0.74841(6)	258(2)
O1	4*c*	0.0690(3)	$^1/_4$	0.82737(17)	376(7)
O2	4*c*	0.3561(3)	$^1/_4$	0.7515(18)	416(8)
O3	8*d*	0.0696(2)	0.13879(19)	0.70885(12)	359(5)
B1	4*c*	0.1765(5)	$^1/_4$	0.1822(3)	272(10)
H1	4*c*	0.131(5)	$^1/_4$	0.246(3)	405(125)
B2	4*c*	0.0526(5)	$^1/_4$	0.1011(3)	292 (11)
H2	4*c*	0.424(4)	$^1/_4$	0.392(2)	161(94)
B3	4*c*	0.4292(5)	$^1/_4$	0.0800(3)	272(10)
H3	4*c*	0.056(5)	$^1/_4$	0.423(2)	335(121)
B4	4*c*	0.3093(5)	$^1/_4$	0.9982(3)	305 (11)
H4	4*c*	0.352(5)	$^1/_4$	0.938(3)	348(12)
B5	8*d*	0.3404(4)	0.1656(3)	0.15604(19)	262(7)
H5	8*d*	0.402(3)	0.114(3)	0.207(17)	225(73)
B6	8*d*	0.3204(4)	0.1127(3)	0.0585(2)	2867(7)
H6	8*d*	0.367(4)	0.022(3)	0.0397(18)	391(91)
B7	8*d*	0.1625(4)	0.1129(3)	0.1222(2)	2770(72)
H7	8*d*	0.111(4)	0.020(3)	0.1449(18)	430(100)
B8	8*d*	0.1430(4)	0.1657(3)	0.0240(2)	316(8)
H8	8*d*	0.075(3)	0.111(3)	0.9801(18)	402(88)

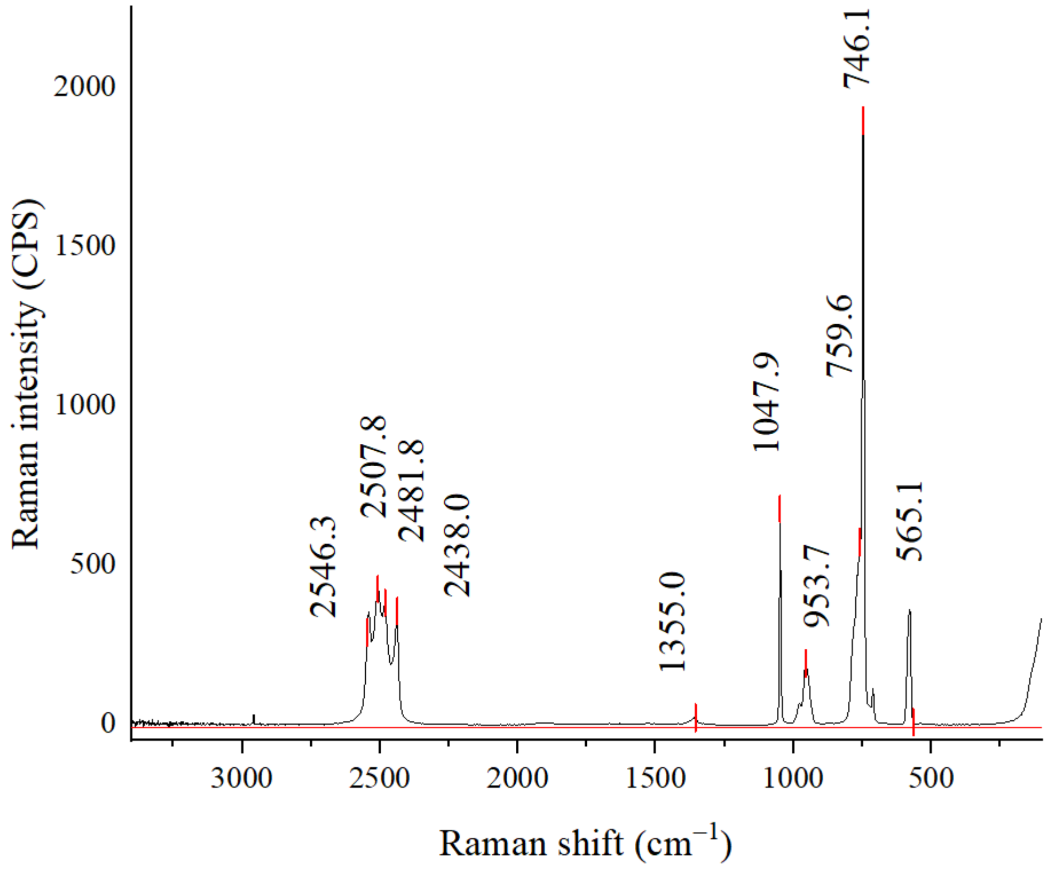

Figure S1. Raman spectrum of $Cs_3[NO_3][B_{12}H_{12}]$ (**I**).

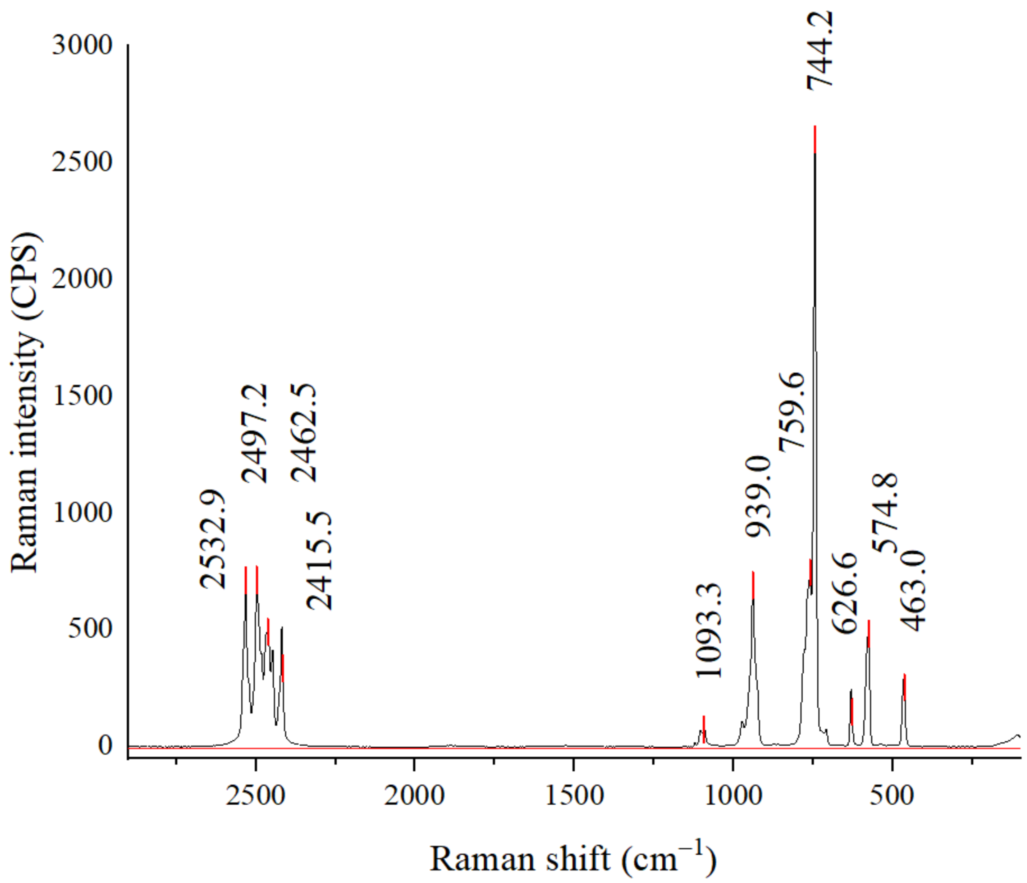

Figure S2. Raman spectrum of $Cs_3[ClO_4][B_{12}H_{12}]$ (**III**).
